# The Effects of a Magic Intervention Program on Cognitive Function and Neurocognitive Performance in Elderly Individuals With Mild Cognitive Impairment

**DOI:** 10.3389/fnagi.2022.854984

**Published:** 2022-04-13

**Authors:** Kuan-Ting Lee, Wei-Li Wang, Wen-Chin Lin, Yi-Ching Yang, Chia-Liang Tsai

**Affiliations:** ^1^Department of Family Medicine, National Cheng Kung University Hospital, Tainan, Taiwan; ^2^Institute of Physical Education, Health and Leisure Studies, National Cheng Kung University, Tainan City, Taiwan; ^3^Department of Family Medicine, Tainan Hospital, Ministry of Health and Welfare, Tainan City, Taiwan; ^4^Department of Family Medicine, College of Medicine, National Cheng Kung University, Tainan City, Taiwan

**Keywords:** magic, mild cognitive impairment, executive function, cognition, event-related potential (ERP)

## Abstract

**Objectives:**

Cognitive training is one of the management options for elderly individuals who suffer from mild cognitive impairment (MCI) and an effective way to improve executive function. This study aimed to evaluate the effectiveness of a magic intervention program as a method of cognitive training in improving cognitive function and neurocognitive performance in this group.

**Methods:**

Twenty-four participants aged 60–80 years with MCI were recruited and randomly assigned to a magic intervention group or a control group. The magic intervention group received a 6-week magic intervention program. The primary endpoints were the scores for the cognitive assessment tests [e.g., Mini-Mental State Examination (MMSE) and Montreal Cognitive Assessment (MoCA)] for general cognitive function. The secondary endpoints were the behavioral [e.g., accuracy and reaction times] and the electroencephalographic [e.g., event-related potential (ERP) P3 amplitudes] performance during the Flanker task to assess attention and inhibitory control. All variables were measured before and after the magic intervention.

**Results:**

The results showed that the 6-week magic intervention significantly improved the MoCA scores in the cognitive assessment tests although no significant pre-post intervention difference was observed in the MMSE scores. In terms of neurocognitive performance, the magic intervention had significantly positive effects on the accuracy, reaction times, and P3 amplitudes when performing the Flanker task.

**Conclusion:**

The results of the present study showed that the 6-week magic intervention had beneficial effects on the cognitive and electrophysiological performance in the elderly subjects with MCI. For such a group, lifestyle intervention programs that encourage participation such as the magic practice and performance may be a viable suggestion to prevent the progression of MCI to Alzheimer’s disease.

## Introduction

Mild cognitive impairment (MCI) is a clinical syndrome and is considered to be a transitional stage from normal aging to dementia (Espinosa et al., 2013). In this stage, patients with MCI show early executive function disorder (Tsai et al., 2016) and deficits in attention (Galvin and Sadowsky, 2012) and inhibitory control (Wylie et al., 2007; Bélanger and Belleville, 2009; Bélanger et al., 2010). Although MCI increases the risk of developing Alzheimer’s disease (AD) by approximately 10–15% each year (Morris et al., 2001), patients with MCI can maintain or even improve their cognitive function through appropriate interventions (Fisk et al., 2003). Indeed, the cognitive reserve hypothesis proposes that trained brains possess greater cognitive reserves than those that are inactive and training can prevent the neuropathological progression of MCI toward AD (Fratiglioni et al., 2004). Fortunately, during the progression of cognitive deterioration, cognitive training has been shown to be a strategy for improving cognitive function as part of healthy aging (Verhaeghen et al., 1992; Nyberg, 2005; Stuss et al., 2007), MCI (Jean et al., 2010; Suzuki et al., 2014; Hill et al., 2017; Peng et al., 2019), and dementia (Woods et al., 2012).

Magic, synonymous with illusion, sorcery, and conjuring, is described as a form of entertainment and a type of hallucinatory science and technology based on human cognitive concepts and the way the human brain interprets five sensory messages (Lam et al., 2017). The magic tricks presented by magicians entertain people by manipulating their attention, perceptions, and awareness and seem to conflict with natural laws (Kawakami and Miura, 2017). When people watch and experience the magic, the brain areas associated with conflict monitoring and error detection [e.g., the left dorsolateral prefrontal cortex (DLPFC) and the anterior cingulate cortex (ACC)] are greatly activated (Parris et al., 2009). Specifically, the DLPFC plays a special role in detecting violations of causality (Parris et al., 2009) and is engaged in the modulation of attention and inhibition during the processing sequences (Osaka et al., 2007). This is because when people evaluate data consistent with plausible theory, they recruit the neural tissue of the parahippocampal gyrus which is related to learning and memory. On the other hand, the ACC and the left DLPFC which deals with error detection and conflict monitoring are recruited when people evaluate data that is inconsistent with plausible theory. More specifically, ACC activation is involved in the detection of conflicts between expected and observed causal relationships, while DLPFC activation is involved in reasoning about observed events and is engaged to resolve these conflicts (Kerns et al., 2004; Fugelsang and Dunbar, 2005; Veen and Carter, 2006). The hierarchical model of the well-being effects of magic developed by Bagienski and Kuhn states that the bottom level is witnessing magic, the second level is discovering magic secrets, the third level is performing magic, and the top level is teaching magic (Bagienski and Kuhn, 2019). Although there is no previous literature directly demonstrating that learning how to perform magic has the same effect as witnessing and feeling the magic, it has been proposed that the higher level of well-being effects are based on and retained from the lower levels (Bagienski and Kuhn, 2020). Thus, watching or performing magic tricks may stimulate DLPFC and enhance executive cognitive functions associated with attention and inhibitory control.

In clinical settings, magic has been applied in occupational and physical therapy, clinical communication, humor therapy, psychotherapy, as well as for dexterity training (Lam et al., 2017). Based on the evidence mentioned above, magic as a method of cognitive training may be able to improve cognitive function and slow the progression of MCI to AD by reversing the early executive function impairments associated with attention and inhibitory control. However, research has yet to be conducted on this issue. Thus, we aimed to determine the effectiveness of a 6-week magic intervention program in improving cognitive function in elderly individuals with MCI. The primary endpoint was the assessment of general cognitive function, and the secondary endpoint was the assessment of neurocognitive performance as well as behavioral performance and electroencephalographic indices related to attention and inhibitory control.

## Materials and Methods

### Participants

We recruited 24 volunteers with MCI aged 60–80 years from Tainan Hospital, Ministry of Health and Welfare. The inclusion criteria (Petersen et al., 1999) were as follows: (1) complaints of memory impairment and confirmed by family members; (2) a Mini-Mental State Examination (MMSE) score of ≥24, no dementia; (3) completely independent on activities of daily living, (4) the 15-item version of the Geriatric Depression Scale (GDS-15) (Sheikh and Yesavage, 1986) score of ≤10, no major depressive disorder; (5) no history of secondary dementia diseases (e.g., neurosyphilis, structural brain lesions, cerebrovascular diseases, etc.); (6) no mental illness, drug abuse or addiction, or use of anti-dementia drugs, (7) normal or corrected to normal vision, and (8) the expected life expectancy of more than 6 months. After baseline evaluation the participants were randomly assigned to either a magic intervention group (*n* = 12) or a control group (*n* = 12). Written informed consent, as approved by the Institutional Ethics Committee of National Cheng Kung University Hospital (IRB No. B-ER-108-332) was obtained from all the participants.

### Sample Size Calculation

The G*Power software (Faul et al., 2007) was used to conduct an *a priori* power analysis of the minimum required sample size. Since no previous studies examined the effect of the magic intervention on cognitive function in older adults with MCI, a study conducted by Peng et al. (2019) which investigated the efficacy of 6-month cognitive training on cognitive function (e.g., MoCA performance) in MCI seniors, was thus referred to determine the value of effect size. The test family was set to “*F*-tests” and “ANOVA: Repeated measures, within-between interaction” was selected for the type of statistical test. The number of intervention modes was set to two with two repetitions [for the 2 (group: magic intervention vs. control) × 2 (time: pre-intervention vs. post-intervention) repeated measures of the primary endpoints]. The type I error probability was set at 0.05, the power level was set to at least 0.80. Afterward, as η*_*p*_*^2^ value was 0.295, the effect size was set accordingly. The estimate of the required total sample size was 12, indicating that 12 participants was the minimum required sample size to obtain a power level of 0.9803.

### Neurocognitive Assessment Tools

#### Cognitive Assessment Tests

##### Mini-Mental State Examination

This test consists of 11 tasks and takes only 5–10 min to complete. It assesses orientation, memory, attention, naming, following verbal and written commands, writing sentences spontaneously, and praxis (i.e., copying a complex polygon) (Folstein et al., 1975). This makes it the most widely used cognitive assessment test in clinical settings.

##### Montreal Cognitive Assessment

This neuropsychological test is designed to rapidly assess mild cognitive impairment. The current version assesses eight different domains of cognitive functioning: visuospatial/executive function, naming, attention, language, abstraction, immediate and delayed memory, and orientation. The total score is 30 points, plus one point if the participant has 12 years or less of education (Nasreddine et al., 2005).

#### Computerized Neurocognitive Assessment

The Flanker task is used to evaluate attention (Luks et al., 2010; Xie et al., 2017), and different types of inhibition (interference inhibition, rule inhibition, and response inhibition) (Xie et al., 2017). It has been shown that elderly people with MCI show cognitive deficits revealed by this task (Wang et al., 2013) in which attentional control accuracy is correlated with the left DLPFC and the ACC, and attentional control reaction time is correlated with the DLPFC (Luks et al., 2010). Specifically, elderly people with MCI exhibited lower accuracy and longer reaction times when performing the Flanker task compared with healthy subjects (Luks et al., 2010; Wang et al., 2013). The cognitive task was programmed with E-prime software (Psychology Software Tools, Inc.)^1^ and was presented as a stimulation array displayed on a computer screen. In this task, the participants were presented with 5 arrows, wherein the target arrow was in the center flanked with two distractor stimuli (flankers) on each side. The directions of the flankers and the target arrow could be congruent (same direction) or incongruent (different directions), and the participants were required to press the left/right button according to the direction of the center arrow.

There were three conditions: (1) the congruent condition (45%), where all the arrows pointed to the same direction, (2) the incongruent condition (45%), where the flankers pointed to the opposite direction of the target arrow, and (3) the neutral condition (10%), where the flankers were represented by “+” symbols. The cognitive task was divided into three blocks, each with 100 randomly ordered stimulus arrays. Each stimulus array had 5 stimuli with the target arrow in the center and the flankers on each side. A participant can rest in between blocks. The trial commenced with a 3-s countdown followed by the appearance of a fixation cross displayed at the center of the computer screen for 1,000 ms and was then replaced by the appearance of the stimulus array for 500 ms to which the participant must respond. If the participant did not respond, a new stimulus array was automatically generated after 2 s. If the participant responded within 2 s, the stimulus array disappeared, and the screen remained blank for 750 ms until a fixed white cross appeared, indicating a new beginning.

#### Event-Related Potential

The EEG was recorded by 18 Ag/AgCl electrodes embedded in a flexible electric cap (F7, F8, F3, F4, Fz, T3, T4, C3, C4, Cz, T5, T6, P3, P4, Pz, O1, O2, Oz; Quik-Cap, Compumedics Neuroscan, Inc., El Paso, TX, United States), with AFz placed in the middle of the forehead as a surface electrode. To monitor eye-movement artifacts, horizontal and vertical bipolar electrooculogram (EOG) data were obtained by placing eye electrodes superolateral to the right and left eye canthi and inferolateral to the left eye canthus.

ERPs are thought to be brain activity potentials generated by an expected event or an environmental stimulus (García-Larrea et al., 2001) and can be used to evaluate cognitive executive function. ERP P3 components are related to attention (Perchet and Garcia-Larrea, 2000; Polich, 2007; Neuhaus et al., 2009) and late general inhibition (Jonkman et al., 2003). Target-locked epochs were created from a 200 ms pre-stimulus baseline to 1,000 ms post-stimulus to calculate the ERP. During the ERP data processing, trials containing response errors and ocular artifacts were removed. The thresholds for the horizontal and vertical bipolar EOG were set at 100 μV. The P3 component was distinguished at three electrodes (Fz, Cz, and Pz). The time window for the mean P3 amplitude was between 300 and 700 ms after the onset of the stimulation.

### Magic Intervention Program

The magic intervention program was taught by a magician who is also a family physician qualified as an instructor by the Ministry of Health and Welfare Prevention and Delay Disability Program. The program was taught for 1.5 h per session twice a week for 6 weeks and each course was assisted by a teaching assistant. The materials used for the magic props were readily available items such as paper, banknotes, coins, rubber bands, paper clips, etc. Daily classes were conducted in small groups with the following routine: first, the magician gave a recap of the previous lesson by inviting the participants to recreate the magic trick presented on the previous class in groups on stage, and assisted them in providing feedback after the performance; second, the magician performed a new magic trick before teaching it to arouse interest and help the participants feel the effects of the magic; and third, which is the most important part, the magician guided the participants to engage in group discussions about possible solutions to the magic trick and to express their ideas in their group. After revealing the secret of the magic trick, the magician showed the participants how to make props which included pasting, drawing, and coloring the materials for the props. Following completion of the props, he demonstrated how to use them in the magic show and guided them on how to extend the original magical effect. Finally, the participants were invited to perform the magic skill they had learned in their group, express their opinions, and provide feedback. As homework, the participants had to review and perform in front of relatives and friends after the course completion. To confirm if they did their homework, the participants were directly asked about their respective experiences of performing magic tricks in front of their relatives and friends before the start of each class. In addition, several participants were randomly selected to perform magic tricks on stage before the class as a review to check if they learned effectively. In the final course, each participant selected a favorite magic trick to perform during the achievement exhibition. All throughout the program, the participants gained abilities in oral expression, memory activities, social interaction, logical thinking, development of fine motor skills and hand-eye coordination, step memory, and most importantly, the ability to think about the principles underlying the secrets of magic and to solve problems. The detailed course contents are shown in Table 1.

**TABLE 1:** The detailed course contents of the magic intervention program.

Week	Class	Content	Class	Content
1	01	Welcome to Hogwarts	02	Bermuda Triangle
2	03	Rocket Launch	04	Magneto
3	05	Four Elements of Life	06	Alice in Wonderland
4	07	Urai Cable Car	08	Lee’s Bank
5	09	Spy J	10	Doraemon’s Bucket
6	11	Naughty Wallet	12	Becoming a Wizard

### Experimental Process

**FIGURE 1:**
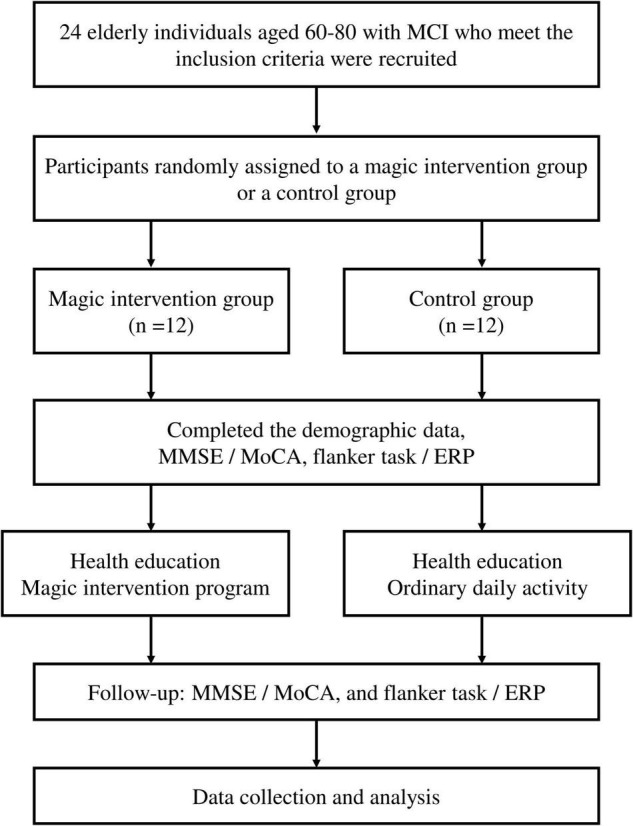
Flowchart of the study.

As illustrated in Figure 1, the participants who met the inclusion criteria were referred to a family physician. The participants were randomly assigned to either a magic intervention group (*n* = 12) or a control group (*n* = 12) after baseline evaluation. Demographic data (e.g., gender, height, weight, past medical and medication history, lifestyle, and social history) were collected using a medical record review and a face-to-face evaluation. The primary endpoints were the scores for the cognitive assessment tests, including the MMSE and the MoCA (a sensitive scale for MCI) that measured general cognitive function. The secondary endpoints were the behavioral performance and electroencephalographic indices related to attention and inhibitory control, as assessed by a Flanker task while EEG data were simultaneously recorded.

All participants received heath education about prevention for dementia once after completing the evaluations mentioned above. The participants in the magic intervention group then received a 6-week magic intervention program. The participants in the control group maintained their original community activities, physical activities, and social activities. After completing the course, all cognitive and EEG tests were conducted again.

### Data Collection and Statistical Analysis

The demographic data were reported as arithmetic mean ± SD. The continuous variables (e.g., age, height, weight, body mass index, years of education, blood pressure, blood indicators that affect cognitive function; Larson, 2016) were analyzed with a Student’s *t*-test. The non-continuous variables (e.g., gender, smoking, RPR/VDRL) were analyzed with a Pearson’s chi-squared test. All data from the cognitive assessment tests (e.g., MMSE/MoCA) and all dependent variables used to analyze the neurocognitive performance, including the accuracy and reaction times for the Flanker task and the ERP P3 amplitudes were analyzed with a repeated-measures analysis of variance (RM-ANOVA). The MMSE scores and MoCA scores were referred to a 2 (group: magic intervention vs. control) × 2 (time: pre-intervention vs. post-intervention) RM-ANOVA. The accuracy and reaction times were referred to a 2 (group: magic intervention vs. control) × 2 (time: pre-intervention vs. post-intervention) × 2 (condition: congruent vs. incongruent) RM-ANOVA, separately. The P3 amplitudes were referred to a 2 (group: magic intervention vs. control) × 2 (time: pre-intervention vs. post-intervention) × 2 (condition: congruent vs. incongruent) × 3 (electrode: Fz vs. Cz vs. Pz) RM-ANOVA. The significance level of the statistical test was set at *p* < 0.05.

## Results

### Demographic Characteristics

As shown in Table 2, there were no significant differences between the magic intervention group and the control group on demographic variables (e.g., age, gender, height, weight, body mass index, years of education, blood pressure, blood indicators) (all *p*s > 0.05). The initial MMSE, MoCA, and GDS-15 scores also revealed non-significant differences across the two groups.

**TABLE 2:** Demographic characteristics of the magic intervention group and control group.

Demographic variables	Magic intervention (*n* = 12)	Control (*n* = 12)	*p*
Age (years)	67.67 ± 4.58	70.00 ± 5.48	0.270
Gender (male/female)	7/5	6/6	0.682
Height (cm)	162.67 ± 8.35	160.16 ± 9.86	0.508
Weight (kg)	70.21 ± 13.46	64.58 ± 10.13	0.259
BMI (kg/m^2^)	26.46 ± 4.48	25.10 ± 2.51	0.370
Education (years)	12.83 ± 3.27	10.00 ± 4.24	0.080
Smoking (%)	8.33	8.33	1.000
SBP(mmHg)	125.08 ± 16.32	13.42 ± 11.39	0.161
DBP(mmHg)	71.75 ± 9.84	71.92 ± 9.34	0.966
MMSE	26.75 ± 2.01	27.33 ± 0.99	0.379
MoCA	23.83 ± 2.41	25.50 ± 2.81	0.133
GDS-15	3.33 ± 2.64	2.42 ± 2.61	0.402
FPG (mg/dL)	98.42 ± 26.65	103.75 ± 18.48	0.575
Hb (g/dL)	13.60 ± 2.61	13.89 ± 0.96	0.720
Cr (mg/dL)	0.897 ± 0.210	0.840 ± 0.178	0.484
ALT (U/L)	29.83 ± 17.98	25.17 ± 11.85	0.461
Vit B12 (pg/mL)	791.42 ± 460.91	737.75 ± 364.20	0.755
Folate (ng/mL)	13.73 ± 4.71	11.59 ± 7.00	0.389
TSH (μIU/mL)	1.77 ± 0.64	2.51 ± 2.26	0.299
RPR/VDRL [(-)%]	100	100	1.000

*Data expressed as Mean ± SD (all ps > 0.05). BMI, body mass index; SBP, systolic blood pressure; DBP, diastolic blood pressure; FPG, fasting plasma glucose; Hb, hemoglobin; Cr, creatinine; ALT, alanine aminotransferase; Vit B12, vitamin B12; TSH, thyroid-stimulating hormone; RPR/VDRL, Rapid Plasma Reagin/Venereal Disease Research Laboratory.*

### Cognitive Performance

**FIGURE 2:**
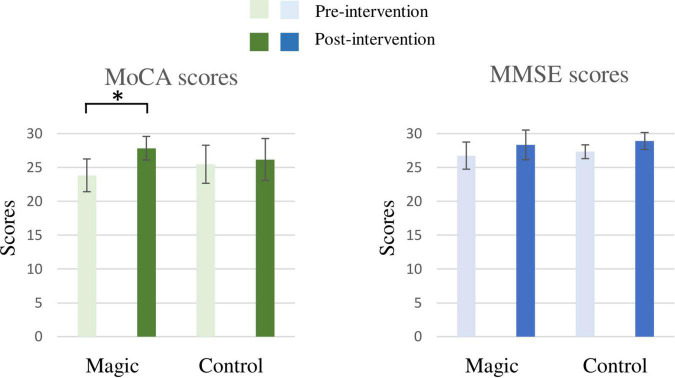
The results of the Mini-Mental State Examination (MMSE) and Montreal Cognitive Assessment (MoCA) of the magic intervention group before and after the magic intervention program and the control group before and after the ordinary daily activity (*p < 0.05).

As illustrated in Figure 2, the RM-ANOVA on the MoCA scores revealed significant main effects of time [*F*_(1, 22)_ = 32.42, *p* < 0.001, η*_*p*_*^2^ = 0.60]. The *post hoc* analyses showed that the post-intervention MoCA scores (27.00 ± 2.60) were higher than the pre-intervention MoCA scores (24.67 ± 2.70) across the two groups. The main effect was superseded by the significant group × time [*F*_(1, 22)_ = 16.54, *p* = 0.001, η*_*p*_*^2^ = 0.43] interaction. The *post hoc* analyses for the group × time interaction revealed that post-intervention MoCA scores (27.83 ± 1.75) were higher than the pre-intervention MoCA scores (23.83 ± 2.41) in the magic intervention group.

In addition, the RM-ANOVA on the MMSE scores revealed significant main effects of time [*F*_(1, 22)_ = 11.63, *p* = 0.003, η*_*p*_*^2^ = 0.35]. The *post hoc* analyses showed that the post-intervention MMSE score (28.63 ± 1.76) was higher than the pre-intervention MMSE score (27.04 ± 1.57) for the two groups. However, there was no significant difference for the group × time interaction.

### Behavioral Performance

**FIGURE 3:**
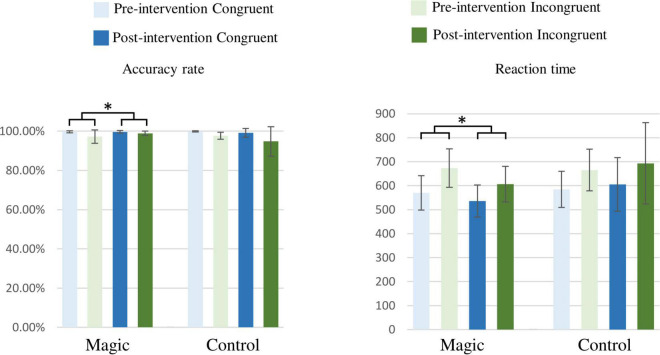
Accuracy rates (*%*) and reaction times (ms) under the congruent and incongruent conditions in the magic intervention group before and after the magic intervention program and in the control group before and after the ordinary daily activity (*p < 0.05).

As illustrated in Figure 3, the RM-ANOVA on the accuracy revealed significant effects of group × time [*F*_(1, 22)_ = 4.95, *p* = 0.037, η*_*p*_*^2^ = 0.18] and condition [*F*_(1, 22)_ = 10.17, *p* = 0.004, η*_*p*_*^2^ = 0.32]. The *post hoc* analyses revealed that in the magic intervention group, the post-intervention accuracy (99.4 ± 1.0%) were higher than the pre-intervention accuracy (98.5 ± 2.6%), and the accuracy in the congruent condition (99.6 ± 1.1%) were higher than in the incongruent one (97.4 ± 4.2%) across the two groups and the two time points.

In addition, the RM-ANOVA on the reaction times revealed significant effects of group × time interaction [*F*_(1, 22)_ = 4.44, *p* = 0.047, η*_*p*_*^2^ = 0.17] and condition [*F*_(1, 22)_ = 101.21, *p* < 0.001, η*_*p*_*^2^ = 0.82]. The *post hoc* analyses showed that in the magic intervention group, post-intervention reaction times (571.31 ± 75.38 ms) were shorter than the pre-intervention reaction times (621.39 ± 88.15 ms) and the reaction times in the incongruent condition (657.51 ± 106.60 ms) were longer than those in the congruent condition (573.84 ± 81.21 ms) across the two groups and the two time points.

### P3 Amplitude

**FIGURE 4:**
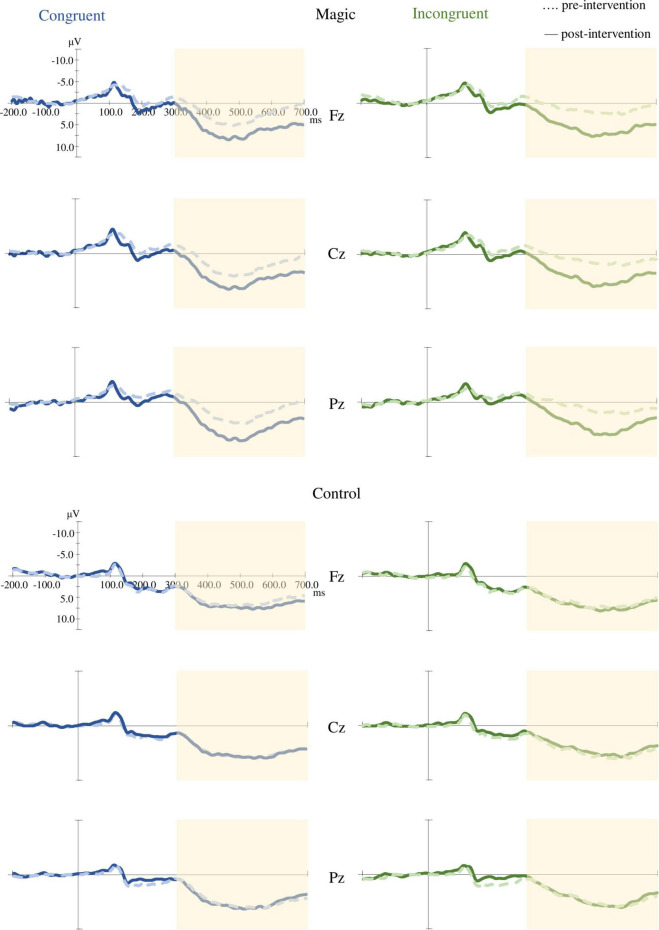
Grand averaged ERP (Fz, Cz, and Pz) under the congruent and incongruent conditions in the magic intervention group before and after the magic intervention program and in the control group before and after the ordinary daily activity.

As illustrated in Figure 4, the RM-ANOVA on the P3 amplitudes revealed significant main effects of time [*F*_(1, 22)_ = 8.46, *p* = 0.008, η*_*p*_*^2^ = 0.28] and condition [*F*_(1, 22)_ = 10.94, *p* = 0.003, η*_*p*_*^2^ = 0.33]. The *post hoc* analyses showed that the post-intervention P3 amplitudes (9.54 ± 4.21 μV) were larger than those in the pre-intervention P3 amplitudes (7.22 ± 5.13 μV) across the two groups, the two conditions, and the three electrodes, and the P3 amplitudes in the incongruent condition (7.91 ± 4.87 μV) were smaller than those in the congruent condition (8.84 ± 4.76 μV) across the two groups, the three electrodes, and the two time points.

Moreover, the RM-ANOVA on the P3 amplitudes indicated significant effects of group × time interaction [*F*_(1, 22)_ = 4.59, *p* = 0.043, η*_*p*_*^2^ = 0.17]. The *post hoc* analyses showed that in the magic intervention group the post-intervention P3 amplitudes (9.41 ± 4.78 μV) were larger than the pre-intervention P3 amplitudes (5.37 ± 5.80 μV).

## Discussion

The present study is the first study to use magic as a cognitive intervention to improve cognitive function and demonstrate its effectiveness in elderly individuals with MCI. In the cognitive assessment tests, the magic intervention program significantly improved the MoCA scores but not the MMSE scores. In terms of neurocognitive performance, the magic intervention had significant beneficial effects on the accuracy, reaction times, and ERP P3 amplitudes when performing the Flanker task.

In the present study, the magic intervention program was a 6-week group training program, consisting of 90-min courses, twice a week (a total of 12 courses). The course contents included oral expression, memory development, social interaction, logical thinking, the development of fine motor and hand-eye coordination, step memory skills, and most importantly, thinking about the principles behind the secrets of magic and attempting to solve problems. Also, in this magic intervention program, the participants mainly experienced three processes. First, the participants were surprised and entertained by the magical effects which allowed them to ponder the possible secrets behind the effects leading the participants to discuss them. Second, the participants made their magic props after they learned the gimmicks. Finally, they were able to perform magic shows after they learned how to use the props. Our study showed that this form of cognitive training can produce significant improvements in the MoCA scores in these patients with MCI. Although a possible learning effect of the same version of the MoCA used for the pre- and post-tests should be taken into account, this effect would have been minimal because past research has shown that there is no significant learning effect when assessing correlation between tests and retests with only a 1-month interval (Nasreddine et al., 2005). The interval between the pre- and post-tests in the present study was approximately 6 weeks, so it is unlikely that the cognitive improvements in the magic intervention group were due to learning effects. In addition, the non-magic-intervention group was also included in the present study, allowing us to rigorously control the learning effect in the pre- and post-test study. Our findings support previous research (Peng et al., 2019) that showed that a 6-month group training program, consisting of 90-min courses every 2 weeks (a total of 12 courses) and with a focus on training the memory, attention, and calculation improved the cognitive functions of elderly Chinese with MCI as revealed by cognitive assessment tests (e.g., MoCA). However, the MMSE scores did not improve significantly in our study, possibly because of its low sensitivity to evaluate MCI (Trzepacz et al., 2015). In addition, the MoCA was shown to be a better screening test than MMSE for the detection of MCI in elderly people over 60 years old (Ciesielska et al., 2016). Therefore, the 6-week magic intervention program can still be considered as an effective way to improve cognitive functions in elderly individuals with MCI.

Among the three processes of the magic intervention program on cognitive function, the most important and most influential one is the first process, which allowed the participants to try explaining the underlying rationale of the magic through personal reflection and group discussion after witnessing it that seemed to violate causality. Compared to a previous study in which fMRI confirmed that magic activated the DLPFC when the participants watched magic videos (Parris et al., 2009), the magic intervention program in the current study would result in a greater activation of the DLPFC. There are two possible reasons: first, while the participants might try to explain the magical effect that violates causal relationship and thus activate their DLPFC, the intensity of the activation was much lower when they watched the magic video than participating in the magic intervention program. In fact, we noted that during the 6 weeks of the magic intervention program, the participants’ ability to explain all or part of the principles of the magical effect improved from the start when they could not explain the principles at all. Second, the magic intervention program provided the participants with plenty of opportunities to perform magic, including performing magic for family and friends at home after each lesson, reviewing the performance at the beginning of each lesson, and having an exhibition at the finale. When performing magic as novices they still felt the conflict of violating the causal relationship, but in order to perform magic well, they had to constantly recall the solution to these conflicts. This process would continue to activate the DLPFC; however, this effect will naturally and gradually decrease as novice magicians become veterans. A study by Danek et al. (2015) showed that the DLPFC is no longer activated when a professional magician watched the magic videos performed by himself. The possible explanation is that professional magicians had practiced these magical techniques for at least 150–200 or more times, and there was no longer any conflict of violation of causality (Danek et al., 2015).

Patients with MCI show early attentional impairment (Galvin and Sadowsky, 2012; Tsai et al., 2016) and deficits in inhibitory control (Wylie et al., 2007; Bélanger and Belleville, 2009; Bélanger et al., 2010). Also, compared with healthy elderly individuals, the MCI cohort exhibited lower accuracy and slower reaction times when performing the Flanker task (Luks et al., 2010; Wang et al., 2013) which corresponded to anomalies in the DLPFC and the ACC (Luks et al., 2010). Previous studies have shown that a 16-week creative expression program consisting of creative storytelling activities of 60 min/session facilitated by a group of professional therapists could shorten the reaction times when performing the auditory oddball paradigm (Zhao et al., 2020). In the present study, the magic intervention program also significantly improved the accuracy and reaction time performance in patients with MCI when performing the Flanker task, suggesting that this cognitive intervention mode facilitated by the magic intervention may enhance the attention and the efficiency of cognitive processing related to inhibitory control in elderly people with MCI (Parris et al., 2009). However, in the present study, it is worth noticing that the beneficial effects on accuracy and reaction times are shown not only in the incongruent condition but also in the congruent one. Indeed, the Flanker task responded to the top-down attentional control system mediated by the ACC/DLPFC, with poorer attentional control as evidenced by the selective decrease in accuracy and longer reaction times under incongruent conditions in contrast to congruent conditions (Luks et al., 2010). The present study shows that the magic intervention program improved both congruent and incongruent conditions simultaneously in the patients with MCI when performing the Flanker task which could mean that magic may have other mechanisms of cognitive stimulation apart from activating DLPFC and ACC. For instance, in addition to the violation of causality, the violation of expectation caused by magic include an element of surprise which can activate the left ventrolateral prefrontal cortex (VLPFC) as opposed to the causal control condition according to a previous study (Parris et al., 2009). This supports the role of VLPFC in dealing with surprising stimuli. The reaction times of patients with neurodegenerative diseases in the Flanker task was not only affected by the ACC-DLPFC network, but also by the temporal-parietal junction (TPJ)-VLPFC network. This may be due to a poor functioning of the top-down attentional control system resulting from neurodegenerative disease, which is further compensated by the bottom-up attentional control system (Luks et al., 2010). When people witness magic, they can detect the tricks through the bottom-up unconscious system although they cannot detect the tricks through the top-down conscious system (Kawakami and Miura, 2017). Thus, it is suggested that magic stimulates not only the ACC/DLPFC mediated top-down attentional control system, which is associated with violation of causality, but also the VLPFC mediated bottom-up attentional control system, which is associated with surprise. As a result, the magic intervention program in this study may have resulted in improved cognitive function by stimulating these brain areas.

Based on previous studies the ERP P3 component has been proposed to be a biomarker involving attention (Perchet and Garcia-Larrea, 2000; Polich, 2007; Neuhaus et al., 2009), late general inhibition (Jonkman et al., 2003; Xie et al., 2017), and memory processing (Polich, 2007), as well as a sensitive indicator of cognitive decline that may help identify early abnormalities in neurophysiological performance (Tsai et al., 2017). Smaller ERP P3 amplitudes were reported in elderly people with MCI when performing the Flanker task (Wang et al., 2013). Past research revealed that a 5-month, twice-a-week, 90 min/session virtual reality museum cognitive training or a learning-based memory training could increase the P3 amplitudes in elderly individuals with MCI (Tarnanas et al., 2014). In addition, previous studies have shown that anodal transcranial direct current stimulation toward the left DLPFC can improve executive function, as evidenced by a significant improvement in reaction times and an increase in ERP P3 amplitudes when performing the Flanker task (Dubreuil-Vall et al., 2019). In the present study, the magic intervention program also significantly enlarged the P3 amplitudes in the patients with MCI. Therefore, the magic intervention program in this study may improve executive function by stimulating the left DLPFC, a brain region associated with attention (Osaka et al., 2007; Luks et al., 2010), inhibition (Osaka et al., 2007), and conflict resolution (Kerns et al., 2004; Fugelsang and Dunbar, 2005; Veen and Carter, 2006). Also, magic may be a cognitive intervention option that can address deficits in attention and inhibition in this group.

Although this is the first study using a magic intervention program as a cognitive stimulation protocol for elderly people with MCI, this study had some limitations. First, the present study only included 24 participants. Future studies with more participants are needed to further evaluate the effectiveness of the magic intervention program. Second, this study only evaluated the short-term effects of a magic intervention program on cognitive and EEG performance. Follow-up research examining the long-term benefits of a magic intervention program is warranted. Third, although direct questioning and pre-session performance were used to assess and enhance the learning outcomes of the participants, there was a lack of quantitative assessment of the intensity and duration of homework. An avenue for future work is to examine the contribution of the homework variables on neurocognitive improvement in patients with MCI. Finally, although we hypothesize that the magic intervention program adopted in the present study improved cognitive function in older adults with MCI by inheriting the cognitive conflict generated by watching magic tricks based on a hierarchical model of the well-being effects of magic developed by Bagienski and Kuhn (2019), such an assumption may be an oversimplification since there are many other items which may also improve cognitive function in the entire magic intervention program, such as oral expression, social interaction, logical thinking, development of fine-motor skills and hand-eye coordination, and step memory. Therefore, follow-up studies may be conducted to evaluate the effects of interventions on specific cognitive aspects so more efficient cognitive training programs can be designed in the future.

## Conclusion

The present study shows that in elderly individuals with MCI, a 6-week magic intervention program produced positive effects on cognitive functions (e.g., MoCA tests) and neurocognitive performance during a cognitive task related to attention and inhibitory control. Thus, a cognitive intervention like the one used in the present study as a lifestyle modification method may be a viable option to prevent the progression of MCI to AD.

The present study is the first study to use magic as a cognitive intervention to improve cognitive function and demonstrate its effectiveness in elderly individuals with MCI. While there is currently no cure for dementia (Perneczky, 2019), measures to delay the progression of MCI into dementia are particularly important. The magic intervention program not only provides an additional intervention option, but it is also more appealing and entertaining for MCI seniors because of its unique appeal. Through this study, the application of magic as a form of cognitive intervention can be further developed in the future.

## Data Availability Statement

The raw data supporting the conclusions of this article will be made available by the authors, without undue reservation.

## Ethics Statement

The studies involving human participants were reviewed and approved by the Institutional Ethics Committee of National Cheng Kung University Hospital. The patients/participants provided their written informed consent to participate in this study.

## Author Contributions

K-TL and W-LW were equally responsible for study design, data collection, data analysis, and writing the manuscript. W-CL was responsible for data collection and data analysis. C-LT and Y-CY were equally responsible for study design, data analysis and manuscript revisions. All authors contributed to the article and approved the submitted version.

## Conflict of Interest

The authors declare that the research was conducted in the absence of any commercial or financial relationships that could be construed as a potential conflict of interest.

## Publisher’s Note

All claims expressed in this article are solely those of the authors and do not necessarily represent those of their affiliated organizations, or those of the publisher, the editors and the reviewers. Any product that may be evaluated in this article, or claim that may be made by its manufacturer, is not guaranteed or endorsed by the publisher.

## Footnotes

1
www.pstnet.com
