# Corrigendum: The Effects of a Magic Intervention Program on Cognitive Function and Neurocognitive Performance in Elderly Individuals With Mild Cognitive Impairment

**DOI:** 10.3389/fnagi.2022.963605

**Published:** 2022-07-13

**Authors:** Kuan-Ting Lee, Wei-Li Wang, Wen-Chin Lin, Yi-Ching Yang, Chia-Liang Tsai

**Affiliations:** ^1^Department of Family Medicine, National Cheng Kung University Hospital, Tainan, Taiwan; ^2^Institute of Physical Education, Health and Leisure Studies, National Cheng Kung University, Tainan City, Taiwan; ^3^Department of Family Medicine, Tainan Hospital, Ministry of Health and Welfare, Tainan City, Taiwan; ^4^Department of Family Medicine, College of Medicine, National Cheng Kung University, Tainan City, Taiwan

**Keywords:** magic, mild cognitive impairment, executive function, cognition, event-related potential (ERP)

In the published article, there were errors. Mean ± standard error was incorrectly used instead of mean ± deviation, the numbers of MMSE scores were misplaced, and the 2nd decimal place of the mean of P3 amplitude in the congruent condition and baseline of intervention group were misplaced.

A correction has been made to Results, Cognitive Performance, Paragraph 1 and 2:

As illustrated in Figure 2, the RM-ANOVA on the MoCA scores revealed significant main effects of time [*F*_(1,22)_ = 32.42, *p* < 0.001, η*p*^2^ = 0.60]. The *post hoc* analyses showed that the post-intervention MoCA scores (27.00 ± 2.60) were higher than the pre-intervention MoCA scores (24.67 ± 2.70) across the two groups. The main effect was superseded by the significant group × time [*F*_(1,22)_ = 16.54, *p* = 0.001, η*p*^2^ = 0.43] interaction. The *post hoc* analyses for the group × time interaction revealed that post-intervention MoCA scores (27.83 ± 1.75) were higher than the pre-intervention MoCA scores (23.83 ± 2.41) in the magic intervention group.

In addition, the RM-ANOVA on the MMSE scores revealed significant main effects of time [*F*_(1,22)_ = 11.63, *p* = 0.003, η_*p*_^2^ = 0.35]. The *post hoc* analyses showed that the post-intervention MMSE score (28.63 ± 1.76) was higher than the pre-intervention MMSE score (27.04 ± 1.57) for the two groups. However, there was no significant difference for the group × time interaction.

A correction has been made to Results, Behavioral Performance, Paragraph 1 and 2:

As illustrated in Figure 3, the RM-ANOVA on the accuracy revealed significant effects of group × time [*F*_(1,22)_ = 4.95, *p* = 0.037, η_*p*_^2^ = 0.18] and condition [*F*_(1,22)_ = 10.17, *p* = 0.004, η_*p*_^2^ = 0.32]. The *post hoc* analyses revealed that in the magic intervention group, the post-intervention accuracy (99.4 ± 1.0%) were higher than the pre-intervention accuracy (98.5 ± 2.6%), and the accuracy in the congruent condition (99.6 ± 1.1%) were higher than in the incongruent one (97.4 ± 4.2%) across the two groups and the two time points.

In addition, the RM-ANOVA on the reaction times revealed significant effects of group × time interaction [*F*_(1,22)_ = 4.44, *p* = 0.047, η_*p*_^2^ = 0.17] and condition [*F*_(1,22)_ = 101.21, *p* < 0.001, η_*p*_^2^ = 0.82]. The *post hoc* analyses showed that in the magic intervention group, post-intervention reaction times (571.31 ± 75.38 ms) were shorter than the pre-intervention reaction times (621.39 ± 88.15 ms) and the reaction times in the incongruent condition (657.51 ± 106.60 ms) were longer than those in the congruent condition (573.84 ± 81.21 ms) across the two groups and the two time points.

A correction has been made to Results, P3 Amplitude, Paragraph 1 and 2:

As illustrated in Figure 4, the RM-ANOVA on the P3 amplitudes revealed significant main effects of time [*F*_(1,22)_ = 8.46, *p* = 0.008, η_*p*_^2^ = 0.28] and condition [*F*_(1,22)_ = 10.94, *p* = 0.003, η_*p*_^2^ = 0.33]. The *post hoc* analyses showed that the post-intervention P3 amplitudes (9.54 ± 4.21 μV) were larger than those in the pre-intervention P3 amplitudes (7.22 ± 5.13 μV) across the two groups, the two conditions, and the three electrodes, and the P3 amplitudes in the incongruent condition (7.91 ± 4.87 μV) were smaller than those in the congruent condition (8.84 ± 4.76 μV) across the two groups, the three electrodes, and the two time points.

Moreover, the RM-ANOVA on the P3 amplitudes indicated significant effects of group × time interaction [*F*_(1,22)_ = 4.59, *p* = 0.043, η_*p*_^2^ = 0.17]. The *post hoc* analyses showed that in the magic intervention group the post-intervention P3 amplitudes (9.41 ± 4.78 μV) were larger than the pre-intervention P3 amplitudes (5.37 ± 5.80 μV).

The authors apologize for this error and state that this does not change the scientific conclusions of the article in any way. The original article has been updated.

## Publisher's note

All claims expressed in this article are solely those of the authors and do not necessarily represent those of their affiliated organizations, or those of the publisher, the editors and the reviewers. Any product that may be evaluated in this article, or claim that may be made by its manufacturer, is not guaranteed or endorsed by the publisher.

